# Phenotypic and Quantitative Characterization of Mast Cells in Cutaneous Melanoma: Correlation with Staging Metrics

**DOI:** 10.3390/cimb47090752

**Published:** 2025-09-12

**Authors:** Grigory Demyashkin, Dmitrii Atiakshin, Kirill Silakov, Vladimir Shchekin, Maxim Bobrov, Olga Abramova, Matvey Vadyukhin, Tatyana Borovaya, Ekaterina Blinova, Petr Shegay, Andrei Kaprin

**Affiliations:** 1 Department of Digital Oncomorphology, National Medical Research Centre of Radiology, 2nd Botkinsky, 3, 125284 Moscow, Russiatbor27@yandex.ru (T.B.);; 2 Laboratory of Histology and Immunohistochemistry, Institute of Translational Medicine and Biotechnology, I.M. Sechenov First Moscow State Medical University (Sechenov University), Trubetskaya st., 8/2, 119048 Moscow, Russia; 3 Research and Educational Resource Center for Immunophenotyping, Digital Spatial Profiling and Ultrastructural Analysis Innovative Technologies, Peoples’ Friendship University of Russia (RUDN University), Miklukho-Maklaya st., 6, 117198 Moscow, Russia; 4 Department of Fundamental Medicine, Institute for Physics and Engineering in Biomedicine, National Research Nuclear University MEPhI, Kashirskoe shosse, 31, 115409 Moscow, Russia; 5 Department of Urology and Operative Nephrology, Peoples’ Friendship University of Russia (RUDN University), Miklukho-Maklaya str.6, 117198 Moscow, Russia

**Keywords:** melanoma, mast cells, tryptase, chymase

## Abstract

**Background**: Mast cells, key effectors of the innate immune system, are known to participate in various stages of tumor progression, including inflammation, angiogenesis, and extracellular matrix remodeling. Their role in melanoma, particularly in relation to Breslow thickness, pT stage, and AJCC staging, remains unclear. This study aims to quantitatively and phenotypically assess mast cell infiltration in cutaneous melanoma at different stages of progression, focusing on Tryptase- and Chymase-positive subtypes. **Methods**: This retrospective multicenter study included 124 patients with cutaneous melanoma (AJCC 8th edition, stages IA–IIIC). Histological sections were stained with hematoxylin and eosin, and mast cells were visualized using toluidine blue and immunohistochemistry with anti-Tryptase and anti-Chymase antibodies. Mast cells were counted manually in intratumoral and peritumoral regions by two independent observers. Quantitative data were analyzed using non-parametric tests and presented as median [Q1–Q3]. **Results**: Histological examination of 124 melanoma samples confirmed typical features of cutaneous melanoma, with nodular melanoma being the most common subtype (68 cases, 54.8%) and the lower extremities identified as the predominant tumor location (47 cases, 37.9%). Toluidine blue staining verified the presence of mast cells in both intratumoral and peritumoral compartments, with the highest density observed in early-stage melanomas. Immunohistochemical analysis identified both Tryptase+ and Chymase+ mast cells. The intratumoral number of Tryptase+ cells declined from 17 [14–19] per HPF at AJCC stage IA to 6 [5–7] per HPF at stage IIIC, while Chymase+ mast cells decreased from 14 [11–16] per HPF to 2 [1–3] per HPF over the same stages. Peritumoral counts also showed a downward trend, although less pronounced. Overall, the most significant reduction was observed in Chymase+ mast cells, suggesting their potential role as markers of melanoma progression. **Conclusions**: This study highlights the dynamic changes in mast cell populations in cutaneous melanoma, with a pronounced decrease in Chymase^+^ mast cells as the tumor progresses. Further research is needed to explore the mechanistic role of mast cells and their phenotypic shifts in melanoma progression.

## 1. Introduction

Cutaneous melanoma remains one of the most aggressive and prognostically unfavorable malignant tumors, despite the advances in modern dermatologic oncology. According to epidemiological studies, its share among all skin malignancies accounts for approximately 20%, and the incidence continues to rise, particularly among fair-skinned populations in regions with high sun exposure [1,2]. Although the introduction of early diagnostic methods and immunotherapy has led to a decrease in mortality, the prognosis for melanoma remains closely linked to the stage of the disease [3].

One of the key factors determining the behavior of melanoma is the tumor microenvironment—a complex system of cells and molecular signals that actively influences tumor growth, angiogenesis, invasion, and metastasis [4]. Its formation involves fibroblasts, endothelial cells, immune cells of both innate and adaptive immunity, as well as extracellular matrix components [5]. In recent years, particular attention has been paid to the role of mast cells—effector elements of the innate immune response, known for their ability to release a wide range of biologically active substances, including histamine, tryptase, chemokines, and cytokines [6,7,8].

Mast cells are traditionally associated with allergic and inflammatory reactions, but their involvement in the pathogenesis of malignant tumors, including melanoma, is gaining increasing significance. Literature data indicate that, in the tumor microenvironment, mast cells are capable of both stimulating tumor growth and invasion by promoting matrix remodeling and angiogenesis, as well as performing protective functions by participating in antigen presentation and mobilizing immune responses [9]. These contradictory effects appear to depend on the tumor stage, its localization, the functional phenotype of the mast cells, and their spatial distribution relative to the tumor focus [10,11,12].

Despite the existence of some experimental and clinical studies, the quantitative characteristics and pathogenic role of mast cells in stages I–III of cutaneous melanoma remain insufficiently studied. Specifically, the patterns of mast cell infiltration in the tumor and peritumoral zones at different stages of disease are poorly understood. The present study aims to analyze the distribution and density of mast cells in the tissues of cutaneous melanoma at stages I–III, as well as to identify possible associations between these parameters and the different staging metrics of the tumor.

## 2. Materials and Methods of Research

### 2.1. Study Design and Setting

This retrospective, multicenter, cohort study was observational and analytical in design and was conducted spanning 2023–2024 years at the National Medical Research Radiological Center (Moscow, Russia). Potential patient cases were initially identified through a search of the electronic medical record systems at both institutions using International Classification of Diseases, 10th Revision (ICD-10) codes corresponding to the primary diagnosis C43—Malignant melanoma of skin.

### 2.2. Participant Selection

The study included data from 124 patients with a confirmed diagnosis of melanoma. Medical records, primarily case histories, were used for analysis. For all patients, anamnesis, clinical manifestations, disease stage according to the American Joint Committee on Cancer (AJCC 8th edition) classification, the nature of surgical and conservative treatments, as well as disease outcomes, were taken into account. Surgical specimens (paraffin blocks) stained with hematoxylin and eosin were also examined, and histological subtypes of melanoma were studied.

Based on the collected data, patient groups were formed corresponding to stages IA, IB, IIA, IIB, IIC, IIIA, IIIB, and IIIC according to the AJCC 8th edition classification.

Inclusion criteria: Cutaneous melanomas of the specified stages without distant metastases (M0); histological subtypes: superficial spreading melanoma (ICD-O: 8743/3; Superficial spreading melanoma), nodular melanoma (ICD-O: 8721/3; Nodular melanoma), desmoplastic melanoma (ICD-O: 8745/3; Desmoplastic melanoma); functional status according to ECOG scale 0–1.

Exclusion criteria: Spitz-type lesions; prior systemic or local antitumor therapy; presence of distant metastases or recurrent disease; synchronous or metachronous multiple primary cancers; infectious diseases; autoimmune disorders.

### 2.3. Histologic Research

All histological specimens were processed and stained in the same pathology laboratory. Serial 2 μm-thick sections were obtained from archival paraffin-embedded tissue blocks of skin. Sections were stained with hematoxylin and eosin (H&E). Toluidine blue staining was also performed to identify mast cells. Histological analysis was performed using a Leica DM2000 light microscope with microphotography capabilities (Leica Microsystems, Wetzlar, Germany), in accordance with standard histopathological criteria.

### 2.4. Immunohistochemical (IHC) Assay

Immunohistochemistry was performed according to standard protocols. Primary antibodies used included anti-Tryptase (Abcam; ab2378, dilution 1:1000) and anti-Chymase (Abcam; ab2377, dilution 1:500). For secondary antibody detection, the HiDef Detec-tion™ HRP Polymer System (Cell Marque, Rocklin, CA, USA) was applied. This two-component system included anti-mouse/rabbit IgG antibodies, horseradish peroxidase (HRP), and DAB chromogen substrate. Cell nuclei were counterstained with Mayer’s hematoxylin.

### 2.5. Morphometric Study

The quantification of Tryptase^+^ and Chymase^+^ mast cells was performed by counting tumor buds in hotspot areas. The assessment was carried out on immunohistochemically stained tissue sections. Initially, areas with the highest mast cell density were identified by scanning the slides at low magnification (×40–100) using QuPath version 5.1. Depending on the size of the lesion, between three and five hotspot areas were selected for counting: three in cases of small lesions and five for larger tumor areas. Mast cells were counted manually in both intratumoral regions and the peritumoral stroma under high-power fields (×400) by two independent observers, with discrepancies resolved by consensus. Images were acquired at ×400 magnification using QuPath V5.1, and the number of Tryptase^+^ and Chymase^+^ cells in each hotspot was recorded. The final mast cells count for each sample was calculated as the average across all selected hotspots. Mast cells located in close proximity to necrotic areas (≈100 µm from the border of necrosis) were excluded from the analysis. This approach was adopted because necrotic tissue lacks a viable microenvironment and is not representative of functional tumor–stroma interactions. Including such regions could artificially lower mast cell counts, especially in advanced stages where necrosis is more common, and would not reflect the biologically relevant distribution of mast cells within viable tumor tissue. In cases of melanin deposition, Tryptase^+^ and Chymase^+^ mast cells were evaluated based on their cellular and nuclear morphology.

### 2.6. Data Collection

Data for this study were systematically extracted from the electronic medical records system. The collected data were then entered into a secure, encrypted Microsoft Excel spreadsheet (Microsoft Excel 2010; Microsoft Corporation, Seattle, WA, USA) for further processing and analysis. The spreadsheet was subsequently reviewed and verified by several co-investigators from the research team, who performed thorough checks to ensure the accuracy and completeness of the dataset before analysis. This multi-step verification process helped minimize errors and ensured the reliability of the data.

### 2.7. Statistical Analysis

Statistical analysis was performed using SPSS software, version 12.0 (IBM Analytics, Armonk, NY, USA). The distribution of quantitative data was assessed with the Shapiro–Wilk test. As the mast cell count data did not follow a normal distribution (*p* < 0.05), non-parametric methods were applied. Intergroup comparisons were conducted using the Mann–Whitney U test, and multiple-group comparisons were performed with the Kruskal–Wallis test followed by post hoc pairwise analysis where appropriate. Quantitative data are presented as median and interquartile range (Me [Q1–Q3]). A *p*-value < 0.05 was considered statistically significant. Data visualization was performed using boxplots, generated in Microsoft Excel, with additional schematic illustrations prepared in BioRender.com (accessed on 7 August 2025).

## 3. Results

### 3.1. Characteristics of Patients

Table 1 summarizes the clinical and pathological characteristics of the patient cohort. A total of 124 individuals diagnosed with cutaneous melanoma were included in the study, with an average age of 63.8 years. Nodular melanoma emerged as the most prevalent histological subtype. The lower extremities represented the most frequent site of tumor involvement, observed in 47 patients. According to the AJCC 8th edition classification, disease stages ranged from IA to IIIC, with the majority of patients falling into advanced stages (IIIA–IIIC).

### 3.2. Histological Patterns

**Figure 1:**
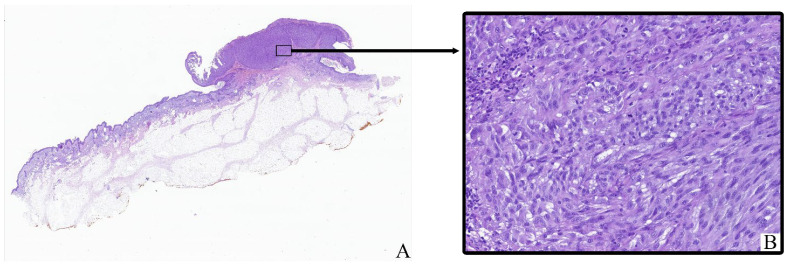
Patient L., male, 55 years. Invasive melanoma, 8743/3 superficial spreading subtype, Breslow thickness 2.1 mm (pT3b). (**A**)—histoscan; (**B**)—tumor. Staining: hematoxylin and eosin, magnification ×400.

All tumor samples from the study cohort (*n* = 124; 100%) exhibited histopathological features consistent with cutaneous melanoma (Figure 1). The neoplastic tissue mainly consisted of irregular clusters, sheets, or fascicles of atypical melanocytic cells infiltrating the dermis and often extending into the subcutaneous fat. Tumor architecture varied between nodular and diffuse patterns depending on the histological subtype.

Most tumor cells displayed marked cytological pleomorphism. The cells were oval, polygonal, or spindle-shaped with sparse to moderate eosinophilic cytoplasm. In many cases, the cytoplasm contained coarse brown pigment granules corresponding to melanin. The nuclei were enlarged, hyperchromatic, and frequently irregular in shape, with prominent nucleoli observed in a significant proportion of tumor cells.

The tumor stroma ranged from fibrotic to moderately inflamed, often containing moderate peritumoral and intratumoral inflammatory infiltrates composed of lymphocytes, plasma cells, and scattered histiocytes. Occasional eosinophils and neutrophils were also present. Some samples showed areas of intercellular edema, vascular congestion, and focal coagulative necrosis, particularly in larger or more deeply invasive tumors.

Ulceration of the overlying epidermis was noted in a subset of cases, often accompanied by reactive epidermal hyperplasia at the periphery. Mitotic figures, including atypical mitoses, were readily identified in most tumors, with mitotic indices varying by stage and histological subtype.

Histological staging according to the 8th edition of the AJCC classification ranged from early superficial involvement (stage IA) to deeply invasive lesions with satellitosis or in-transit metastases (stage IIIC). In lower-stage tumors (e.g., IA–IIB), the neoplastic process was mostly confined to the dermis, whereas advanced-stage tumors (IIIC) demonstrated extensive infiltration into the subcutis, vascular or lymphatic invasion, and in some cases, involvement of adjacent structures.

### 3.3. Toluidine Blue Assay

**Figure 2:**
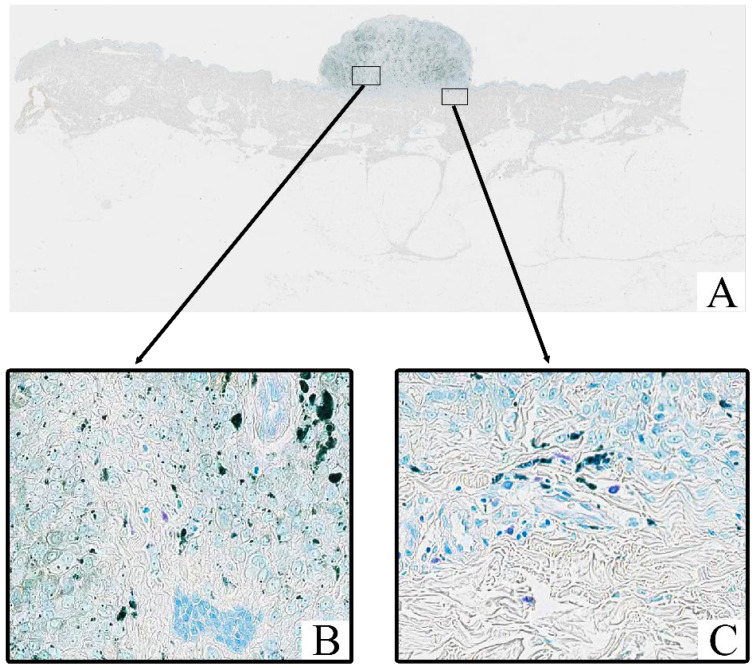
Patient I., female, 88 years. Invasive melanoma, 8721/3 nodular subtype, Breslow thickness 1.8 mm (pT2a). (**A**)—histoscan; (**B**)—intratumoral tissue; (**C**)—peritumoral tissue. Staining: Toluidine Blue, magnification ×400.

All tumor samples from the study cohort (*n* = 124; 100%) were subjected to Toluidine Blue staining, which highlighted the presence of mast cells within the tumor stroma. In all samples, mast cells exhibited a distinct purple coloration, indicative of metachromatic staining of their granules (Figure 2). Toluidine Blue staining revealed that the granules of mast cells were prominently stained in purple, with a characteristic metachromatic shift. These cells were predominantly located in the tumor stroma, particularly in areas with peritumoral and intratumoral inflammatory infiltrates.

### 3.4. Immunohistochemical Assay

Immunohistochemical staining for Tryptase and Chymase revealed the presence of Tryptase^+^ and Chymase^+^ mast cells within both the intratumoral and peritumoral compartments of cutaneous melanoma samples. These cells were predominantly scattered as individual elements or in small clusters, with a tendency to localize near areas of dense tumor cell infiltration and stromal remodeling.

**Figure 3:**
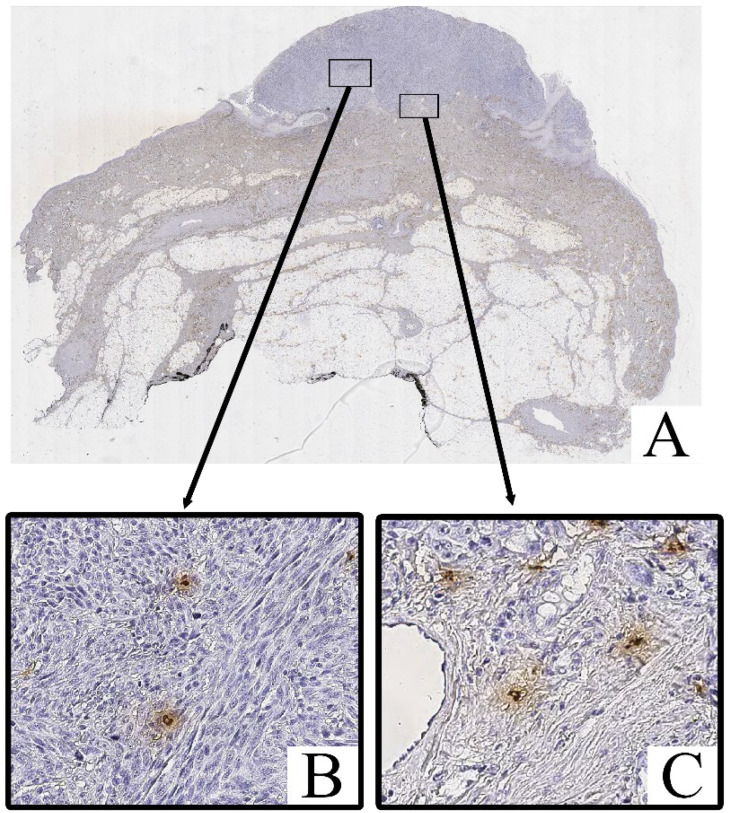
Patient K., male, 43 years. Invasive melanoma, 8721/3 nodular subtype, Breslow thickness 4.6 mm (pT4b). (**A**)—histoscan; (**B**)—intratumoral tissue; (**C**)—peritumoral tissue. Immunohistochemical reaction with antibodies to Tryptase, magnification ×400.

**Figure 4:**
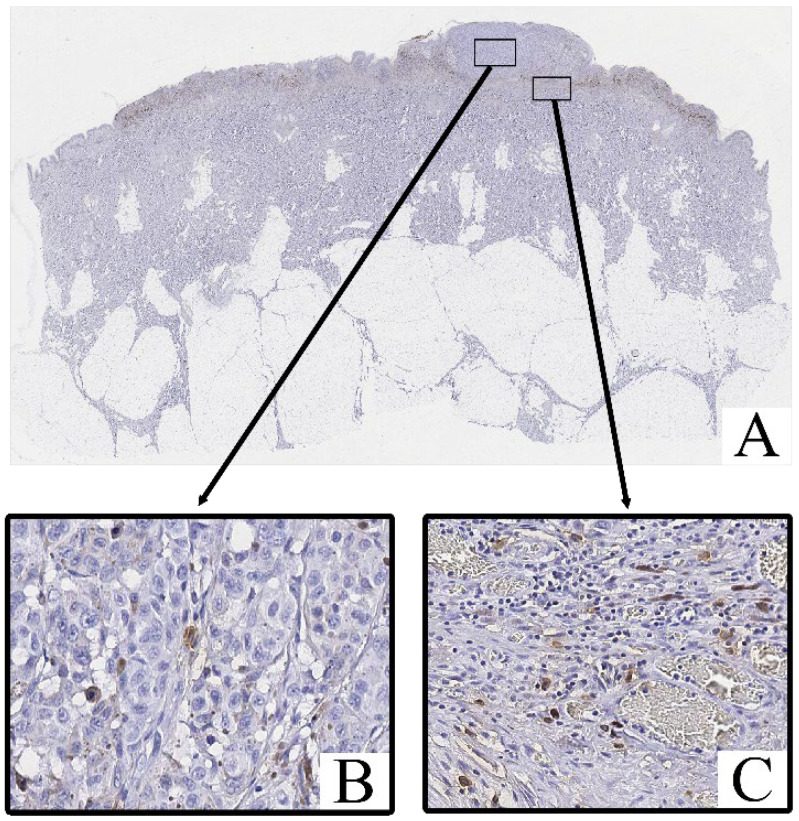
Patient G., male, 53 years. Invasive melanoma, 8743/3 superficial spreading subtype, Breslow thickness 1.6 mm (pT2b). (**A**)—histoscan; (**B**)—intratumoral tissue; (**C**)—peritumoral tissue. Immunohistochemical reaction with antibodies to Chymase, magnification ×400.

Both Tryptase^+^ Chymase^+^ and mast cells exhibited strong cytoplasmic immunoreactivity, with their granules staining intensely in the cytoplasm (Figure 3 and Figure 4). These cells displayed an irregular, oval, or spindle-shaped morphology, with large, hyperchromatic nuclei. The staining of Tryptase was observed both within the tumor parenchyma and in the surrounding stroma, with a higher concentration of these cells in more invasive regions of the tumor.

Mast cell infiltration was identified both within the tumor (intratumoral) and in the surrounding tissue (peritumoral), with a notable increase in density in the peritumoral zone.

In contrast, the non-tumorous skin adjacent to the melanoma exhibited only isolated Tryptase^+^ and chymase^+^ mast cells, primarily located around blood vessels or in areas with increased inflammatory activity. Chymase^+^ mast cells were less frequent in these regions, with Tryptase^+^ mast cells being slightly more common, but both were significantly less abundant compared to the tumor tissue.

### 3.5. Quantitative Analysis of Mast Cells According to Breslow Thickness

**Figure 5:**
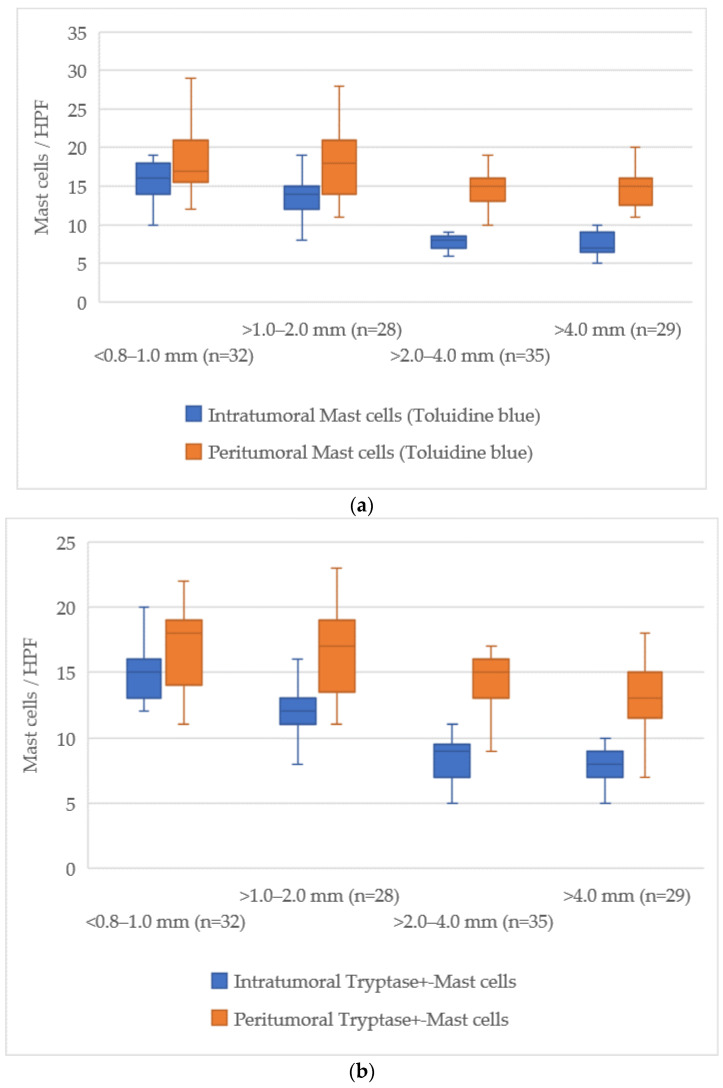

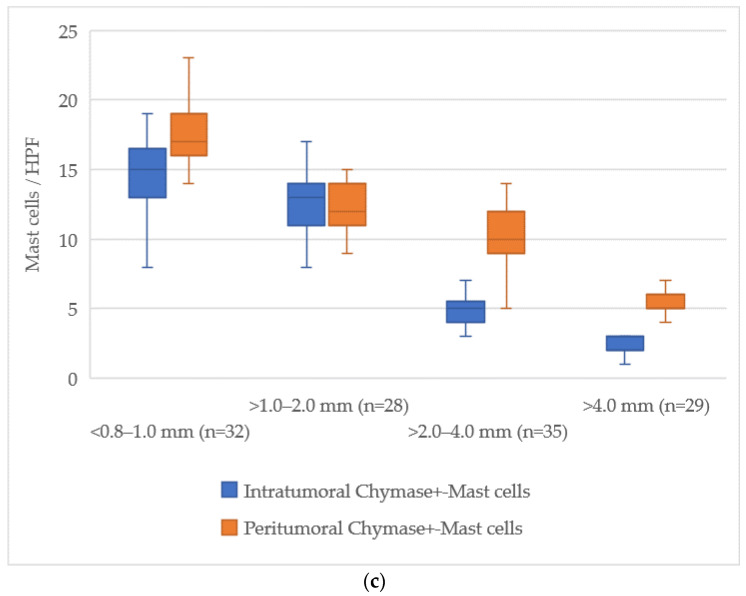
Distribution of mast cell counts by Breslow (*p* < 0.05). (**a**)—toluidine blue; (**b**)—Tryptase; (**c**)—Chymase.

The examination of mast cell markers relative to Breslow thickness revealed a distinct reduction in the number of mast cells both within the tumor and in the surrounding tissues as the tumor thickness increased. The average values of mast cells across different zones are illustrated in Figure 5.

At a tumor thickness of less than 1.0 mm (<0.8–1.0 mm), the mean number of intratumoral mast cells stained with Toluidine Blue was 15 [13–18], while for tumors thicker than 4.0 mm, this value dropped to 7 [6–9], indicating an absolute decrease of about 8 cells. The peritumoral zone exhibited a similar trend, with a decrease from 18 [16–20] at less than 1.0 mm to 14 [13–16] at more than 4.0 mm, reflecting a decrease of about 4 cells.

For Tryptase-positive mast cells, the number in the intratumoral zone decreased from 15 [13–17] cells in tumors with a thickness less than 1.0 mm to 8 [7–9] cells in tumors thicker than 4.0 mm, showing a reduction of about 7 cells. In the peritumoral zone, this reduction was from 17 [15–19] cells to 14 [13–16] cells, corresponding to a reduction of about 3 cells.

Chymase-positive mast cells exhibited the most notable decline in the intratumoral zone, where the number decreased from 14 [12–16] in tumors with a thickness of less than 1.0 mm to 2 [1–3] in those greater than 4.0 mm, representing a reduction of about 12 cells. Similarly, in the peritumoral area, the count reduced from 18 [16–20] to 6 [5–7] cells, a reduction of about 12 cells.

This data shows a progressive reduction in the number of mast cells both within the tumor and in the surrounding tissue, with the most significant decline occurring in chymase-positive mast cells in the intratumoral zone.

### 3.6. Quantitative Analysis of Mast Cells According to pT Stage

**Figure 6:**
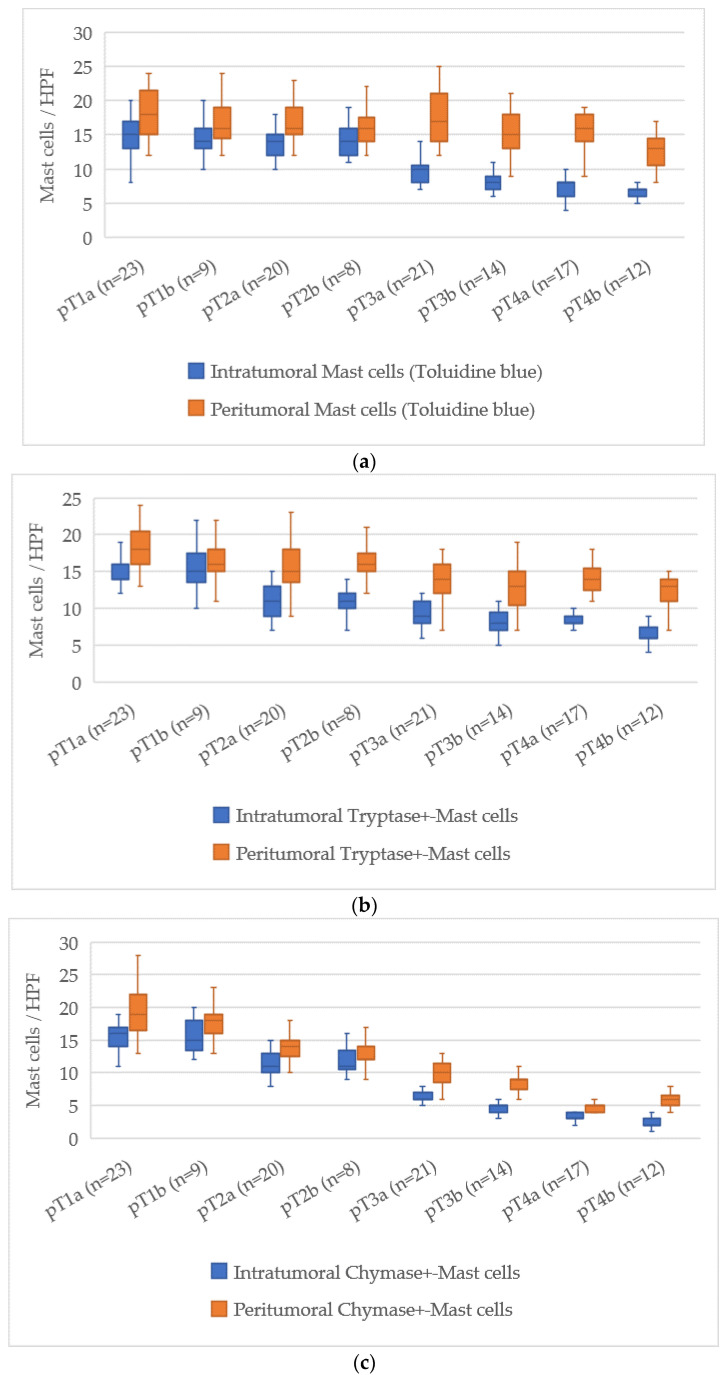
Distribution of mast cell counts by tumor thickness according to pT stage (*p* < 0.05). (**a**)—toluidine blue; (**b**)—Tryptase; (**c**)—Chymase.

The assessment of mast cell markers in relation to the pT stage of cutaneous melanoma demonstrated a clear tendency for mast cell numbers to decrease as the tumor stage advanced. The average mast cell counts for each stage are presented in Figure 6.

At pT1a, the average number of intratumoral mast cells stained with Toluidine Blue was 15 [13–18], which decreased to 7 [6–8] at pT4b, corresponding to a reduction of about 8 cells. For the peritumoral zone, the count reduced from 18 [16–20] at pT1a to 14 [13–16] at pT4b, reflecting a reduction of about 4 cells.

For Tryptase-positive mast cells, the count in the intratumoral area dropped from 15 [13–17] at pT1a to 6 [5–8] at pT4b, a reduction of about 9 cells. In the peritumoral zone, Tryptase-positive cells decreased from 18 [16–20] at pT1a to 13 [12–15] at pT4b, with a decrease of 5 cells.

Chymase-positive mast cells showed the most significant decline in the intratumoral zone. At pT1a, their number was 16 [14–18], while at pT4b it decreased to 2 [1–3], reflecting a reduction of about 14 cells. In the peritumoral area, this reduction was from 18 [16–20] at pT1a to 6 [5–7] at pT4b, a reduction of about 12 cells.

Overall, the pT staging scale revealed a steady reduction in both intratumoral and peritumoral mast cell counts, with the most pronounced decrease seen in Chymase-positive mast cells. This reduction may suggest an evolving role of mast cells in the progression of melanoma, with their number declining as the disease reaches more advanced stages.

### 3.7. Quantitative Analysis of Mast Cells According to AJCC Stage

**Figure 7:**
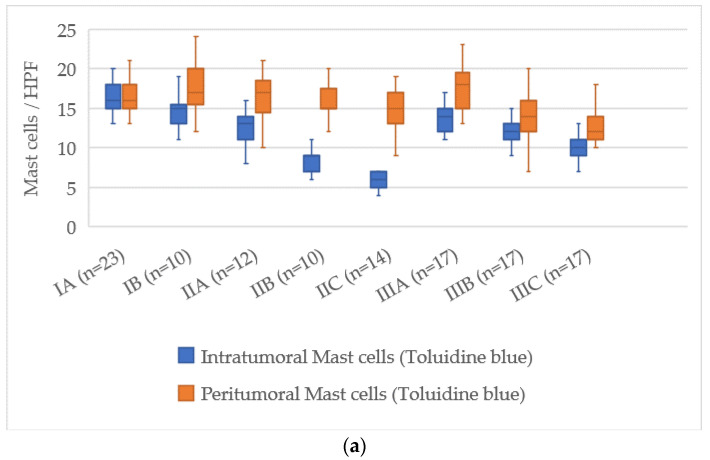

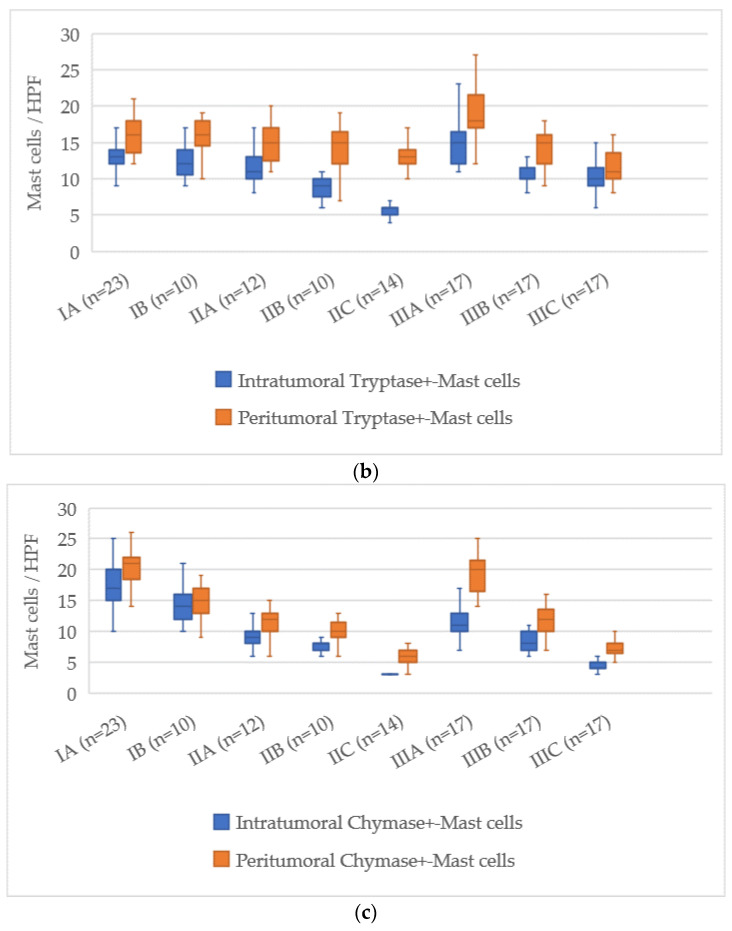
Correlation of mast cell count with tumor thickness according to AJCC stage (*p* < 0.05). (**a**)—toluidine blue; (**b**)—Tryptase; (**c**)—Chymase.

The assessment of mast cell markers in relation to the AJCC stage of cutaneous melanoma showed a clear pattern of decreasing mast cell numbers as the disease progressed. The distributions of mast cell counts for each stage are illustrated in Figure 7.

At AJCC stage IA, the number of intratumoral mast cells stained with Toluidine Blue was 16 [14–18], which decreased to 7 [6–8] at stage IIIC, reflecting a reduction of about 9 cells. In the peritumoral zone, the values decreased from 18 [16–20] at stage IA to 14 [13–16] at stage IIIC, with a reduction of about 4 cells.

For Tryptase-positive mast cells, the count in the intratumoral zone at stage IA was 17 [15–19], and at stage IIIC it dropped to 6 [5–7] cells, corresponding to a reduction of about 11 cells. In the peritumoral area, the number decreased from 17 [15–19] at stage IA to 13 [12–15] at stage IIIC, with a reduction of about 4 cells.

Chymase-positive mast cells displayed the most considerable reduction, particularly in the intratumoral zone. At AJCC stage IA, their number was 14 [12–16], and at stage IIIC it decreased to 2 [1–3], representing a reduction of about 12 cells. In the peritumoral zone, the count dropped from 20 [18–22] at stage IA to 7 [6–8] at stage IIIC, reflecting a decrease of about 13 cells.

Thus, the AJCC staging system revealed a consistent decline in both intratumoral and peritumoral mast cells, with the most pronounced reduction occurring in Chymase-positive mast cells. This may indicate a shift in the role of mast cells during melanoma progression as the disease advances to later stages.

## 4. Discussion

The results demonstrated a consistent decrease in mast cell numbers with increasing tumor thickness according to Breslow and with more advanced stages based on the pT and AJCC classifications. Special attention was given to cells positive for serine proteases—Tryptase and Chymase—which are important functional markers of mast cell activation. The results demonstrate a consistent decrease in mast cell numbers with increasing tumor thickness according to Breslow and at more advanced stages based on the pT and AJCC classifications.

Mast cells, as effector cells of the innate immune system, exhibit high functional plasticity and participate in the regulation of inflammation, angiogenesis, extracellular matrix remodeling, and immune responses [13]. Within the tumor microenvironment, they can perform both protumorigenic and antitumorigenic functions, depending on local signals, disease stage, and phenotypic characteristics [14]. Among the key mediators secreted by mast cells, serine proteases—Tryptase and Chymase—play a prominent role, each reflecting a distinct functional profile of the cell. Tryptase-positive mast cells are primarily involved in tissue remodeling, fibroblast activation, and the stimulation of matrix metalloproteinase expression, contributing to tumor invasion and the formation of fibrotic stromal components [15]. In contrast, Chymase-positive mast cells possess more pronounced angiogenic and immunoregulatory activities: they can activate TGF-β, promote neovascularization, and contribute to the creation of an immunosuppressive microenvironment favorable for tumor progression [16]. Thus, the decrease in the number of Chymase-positive cells during the progression to later stages of melanoma may suggest a reduction in specific mechanisms of local immune suppression, whereas the persistence of Tryptase-positive cells may indicate continued support for the inflammatory and remodeling background of the tumor.

At early stages of melanoma, the highest number of mast cells, especially Chymase-positive ones, was recorded, which may reflect their active role in the initial stages of tumor remodeling and inflammatory responses [17]. The reduction in their numbers at later stages may be related to the depletion of regenerative resources, a shift in the profile of signaling molecules in the tumor, or the relocation of key immunosuppressive functions to other cellular elements of the tumor microenvironment, such as TAMs or Tregs [18].

A particularly pronounced reduction in Chymase-positive mast cells compared to Tryptase-positive cells, both in the intratumoral and peritumoral zones, may indicate differences in their functions and sensitivity to changes in the microenvironment. In the context of tumor progression, accompanied by hypoxia, acidosis, and vascular destruction, the survival and functional activity of Chymase-positive cells may be impaired [19]. At the same time, Tryptase-positive cells likely play a more stable role in maintaining the inflammatory and fibrotic background, especially in the peritumoral zone [15].

Importantly, the observed decrease affected not only Chymase-positive but also Tryptase-positive mast cells. Since Tryptase is a potent promoter of angiogenesis, extracellular matrix remodeling, and fibroblast activation, its reduction suggests attenuation of several pro-tumorigenic mechanisms in advanced melanoma [20]. Thus, the overall decline in mast cell numbers may not be limited to the loss of immunosuppressive and regulatory functions, but could also reflect a broader disruption of stromal support and microenvironmental integrity. This dual interpretation underscores the functional heterogeneity of mast cells and suggests that their role in melanoma progression is more complex than a simple shift toward or away from immune suppression.

Comparison with the literature confirms the observed trend of decreasing mast cell density in malignant tumors with high invasiveness. Similar results have been obtained in studies of mast cells in breast, prostate, and gastrointestinal tumors, where their numbers and activity varied depending on tumor stage and aggressiveness [21,22,23]. However, a number of studies indicate a potential association between high mast cell density at early stages and a favorable prognosis [24,25].

Further perspectives lie in the study of molecular signaling pathways regulating the differentiation and migration of mast cells into tumor tissue, such as the SCF/c-Kit axis, as well as the use of multiplex immunohistochemistry and spatial transcriptomics to identify functional mast cell subpopulations in the tumor microenvironment [26].

Thus, the observed decrease in mast cell numbers, especially Chymase-positive cells, at later stages of melanoma may suggest changes in their role during tumor progression and a possible weakening of their participation in the local immune response. These findings highlight the need for further research to clarify the functional heterogeneity of mast cells and to explore their potential role as prognostic biomarkers and therapeutic targets.

This study has certain limitations that should be acknowledged. It relied on histopathological staging parameters (Breslow thickness, pT, and AJCC stage) as surrogates of disease progression. Clinical outcome data, such as progression-free survival or overall survival, were not available, which restricts conclusions regarding prognostic value. These limitations suggest that our results should be regarded as hypothesis-generating and highlight the need for larger, prospective studies with integrated survival analyses to further validate and expand upon our findings.

## 5. Conclusions

The results of this study suggest that with increasing stages of cutaneous melanoma, there is a decrease in the number of mast cells both in the intratumoral and peritumoral zones, with the most pronounced reduction observed in Chymase-positive cells at later stages of the disease. Importantly, the observed decrease affected not only Chymase-positive but also Tryptase-positive mast cells. Since Tryptase is a potent promoter of angiogenesis, extracellular matrix remodeling, and fibroblast activation, its reduction may indicate attenuation of certain pro-tumorigenic mechanisms. Thus, the overall decline in mast cell numbers could reflect both a weakening of immunosuppressive control and a broader disruption of stromal support and microenvironmental integrity in advanced melanoma.

These findings highlight the need for further research to clarify the functional heterogeneity of mast cells and to explore their potential role as prognostic biomarkers and therapeutic targets. Future studies utilizing multiplex immunohistochemistry and progression-free survival analysis are warranted to better understand the associations between mast cell subsets and melanoma progression. Such investigations may help to determine whether the decline in Chymase-positive mast cells is linked to the loss of their immunosuppressive activity at later stages of the disease and could potentially inform the development of personalized therapeutic strategies targeting the tumor microenvironment.

## Figures and Tables

**Table 1:** Clinicopathological Characteristics of the Patients.

Characteristic	*n*	%
Age (range)	29–88 years	N/A
Mean age	63.8 years	N/A
Gender		
Male	53	42.7%
Female	71	57.3%
Histological type		
Superficial spreading melanoma	46	37.1%
Nodular melanoma	68	54.8%
Desmoplastic melanoma	10	8.1%
Breslow thickness		
<0.8–1.0 mm	32	25.8%
>1.0–2.0 mm	28	22.6%
>2.0–4.0 mm	35	28.2%
>4.0 mm	29	23.4%
Clark’s level of invasion		
I	16	12.7%
II	25	19.8%
III	24	19.0%
IV	48	38.9%
V	11	9.5%
Primary tumor localization		
Head and neck	19	15.3%
Trunk	29	23.4%
Upper extremities	29	23.4%
Lower extremities	47	37.9%
Pathological T category (pT)		
pT1a	23	18.5%
pT1b	9	7.3%
pT2a	20	16.1%
pT2b	8	6.5%
pT3a	21	16.9%
pT3b	14	11.3%
pT4a	17	13.7%
pT4b	12	9.7%
AJCC 8th Edition Stage		
IA	23	18.5%
IB	10	8.1%
IIA	12	9.7%
IIB	14	11.3%
IIC	14	11.3%
IIIA	17	13.7%
IIIB	17	13.7%
IIIC	17	13.7%

## Data Availability

The study did not generate publicly available archival data.

## References

[1] Arnold M., Singh D., Laversanne M., Vignat J., Vaccarella S., Meheus F., Cust A.E., de Vries E., Whiteman D.C., Bray F. (2022). Global Burden of Cutaneous Melanoma in 2020 and Projections to 2040. JAMA Dermatol..

[2] Maghfour J., Li P., Piontkowski A., Ozog D.M., Mi Q.S., Veenstra J. (2023). Melanoma Incidence and Mortality: Exploring the Impact of Regional UV Radiation and Socioeconomic Status in the Context of Breslow Thickness. J. Am. Acad. Dermatol..

[3] Didier A.J., Nandwani S., Watkins D., Fahoury A.M., Campbell A., Craig D.J., Vijendra D., Parquet N. (2024). Patterns and trends in melanoma mortality in the United States, 1999–2020. BMC Cancer.

[4] Kharouf N., Flanagan T.W., Hassan S.-Y., Shalaby H., Khabaz M., Hassan S.-L., Megahed M., Haikel Y., Santourlidis S., Hassan M. (2023). Tumor Microenvironment as a Therapeutic Target in Melanoma Treatment. Cancers.

[5] Mazurkiewicz J., Simiczyjew A., Dratkiewicz E., Ziętek M., Matkowski R., Nowak D. (2021). Stromal Cells Present in the Melanoma Niche Affect Tumor Invasiveness and Its Resistance to Therapy. Int. J. Mol. Sci..

[6] Guo X., Liu R. (2023). Role of mast cells activation in the tumor immune microenvironment and immunotherapy of cancers. Eur. J. Pharmacol..

[7] Shi S., Ye L.Y., Yu X., Jin K., Wu W. (2022). Focus on mast cells in the tumor microenvironment: Current knowledge and future directions. Biochim. Biophys. Acta Rev. Cancer.

[8] Saxena S.P., Singh A.K., Singh P. (2020). Tumor associated mast cells: Biological roles and therapeutic applications. Anat. Cell Biol..

[9] Segura-Villalobos D., Ramírez-Moreno I.G., Martínez-Aguilar M., Ibarra-Sánchez A., Muñoz-Bello J.O., Anaya-Rubio I., Padilla A., Macías-Silva M., Lizano M., González-Espinosa C. (2022). Mast Cell–Tumor Interactions: Molecular Mechanisms of Recruitment, Intratumoral Communication and Potential Therapeutic Targets for Tumor Growth. Cells.

[10] Majorini M.T., Cancila V., Rigoni A., Botti L., Dugo M., Triulzi T., De Cecco L., Fontanella E., Jachetti E., Tagliabue E. (2020). Infiltrating Mast Cell-Mediated Stimulation of Estrogen Receptor Activity in Breast Cancer Cells Promotes the Luminal Phenotype. Cancer Res..

[11] Hou Y., Wang Q., Su L., Zhu Y., Xiao Y., Feng F. (2023). Increased tumor-associated mast cells facilitate thyroid cancer progression by inhibiting CD^8+^ T cell function through galectin-9. Braz. J. Med. Biol. Res..

[12] Ribatti D. (2024). New insights into the role of mast cells as a therapeutic target in cancer through the blockade of immune checkpoint inhibitors. Front. Med..

[13] Komi D.E.A., Wöhrl S., Bielory L., Bielory L. (2020). Mast Cell Biology at Molecular Level: A Comprehensive Review. Clin. Rev. Allergy Immunol..

[14] Noto C.N., Hoft S.G., DiPaolo R.J. (2021). Mast Cells as Important Regulators in Autoimmunity and Cancer Development. Front. Cell Dev. Biol..

[15] Komi D.E.A., Redegeld F.A. (2020). Role of Mast Cells in Shaping the Tumor Microenvironment. Clin. Rev. Allergy Immunol..

[16] Nishimura H., Jin D., Kinoshita I., Taniuchi M., Higashino M., Terada T., Takai S., Kawata R. (2023). Increased Chymase-Positive Mast Cells in High-Grade Mucoepidermoid Carcinoma of the Parotid Gland. Int. J. Mol. Sci..

[17] Takai S., Jin D. (2022). Pathophysiological Role of Chymase-Activated Matrix Metalloproteinase-9. Biomedicines.

[18] Lichterman J.N., Reddy S.M. (2021). Mast Cells: A New Frontier for Cancer Immunotherapy. Cells.

[19] Audero M.M., Prevarskaya N., Fiorio Pla A. (2022). Ca^2+^ Signalling and Hypoxia/Acidic Tumour Microenvironment Interplay in Tumour Progression. Int. J. Mol. Sci..

[20] Ribatti D. (2024). Tryptase and tumor angiogenesis. Front. Oncol..

[21] Hanes M.R., Giacomantonio C.A., Marshall J.S. (2021). Mast Cells and Skin and Breast Cancers: A Complicated and Microenvironment-Dependent Role. Cells.

[22] Zadvornyi T., Lukianova N., Borikun T., Tymoshenko A., Mushii O., Voronina O., Vitruk I., Stakhovskyi E., Chekhun V. (2022). Mast cells as a tumor microenvironment factor associated with the aggressiveness of prostate cancer. Neoplasma.

[23] Shu F., Yu J., Liu Y., Wang F., Gou G., Wen M., Luo C., Lu X., Hu Y., Du Q. (2025). Mast cells: Key players in digestive system tumors and their interactions with immune cells. Cell Death Discov..

[24] Fan F., Gao J., Zhao Y., Wang J., Meng L., Ma J., Li T., Han H., Lai J., Gao Z. (2023). Elevated Mast Cell Abundance is Associated with Enrichment of CCR2^+^ Cytotoxic T cells and Favorable Prognosis in Lung Adenocarcinoma. Cancer Res..

[25] Mo S., Zong L., Chen X., Chang X., Lu Z., Yu S., Chen J. (2021). Supplementary Material for: High Mast Cell Density Predicts a Favorable Prognosis in Patients with Pancreatic Neuroendocrine Neoplasms.

[26] Annese T., Tamma R., Bozza M., Zito A., Ribatti D. (2022). Autocrine/Paracrine Loop Between SCF^+^/c-Kit^+^ Mast Cells Promotes Cutaneous Melanoma Progression. Front. Immunol..

